# Clinical management of finger joint capsulitis/synovitis in a rock climber

**DOI:** 10.3389/fspor.2023.1185653

**Published:** 2023-05-25

**Authors:** Jared Vagy

**Affiliations:** Division of Biokinesiology and Physical Therapy, University of Southern California, Los Angeles, CA, United States

**Keywords:** physical therapy, finger pain, joint capsulitis, joint synovitis, rock climbing

## Abstract

This case study presents a 23-year-old male recreational rock climber, who climbed an average of 3–4 times per week and presented with finger joint capsulitis/synovitis after increasing his climbing intensity and training from moderate to high over 6 months, which led up to injury. During the exam, the diagnosis was ruled in with clinical orthopedic testing. Additional movement analyses revealed improper gripping mechanics contributing to asymmetric finger loading. A comprehensive rehabilitation program was developed based on the concept of a progressive framework that included unloading of the affected tissues, increasing mobility, improving muscle performance, and correcting suboptimal climbing movements. After 6 weeks, the climber's pain 24 h after climbing, which was rated on a visual analog pain scale (VAS), decreased from 5.5/10 to 1.5/10 and 0/10 at the 12-month follow-up. His patient-specific functional scale improved from 0% at the initial evaluation to 43% after 6 weeks and to 98% after 12 months. His sports-specific disabilities of the arm, shoulder, and hand improved from 69% to 34% to 6% during the initial evaluation, 6-week follow-up, and 12-month discharge. He made a full recovery to his previous grade of V8 bouldering. This is the first case study of its kind to provide a rehabilitation framework for the management of finger joint capsulitis/synovitis in a rock climber.

## Introduction

Rock climbing is a sport that imposes considerable physical demands on the hands and fingers, which play a crucial role in gripping and holding onto the rock while climbing. As a result, the fingers are particularly susceptible to injuries due to the repeated stress and strain placed. Three of the most common finger injuries in rock climbers are injury to the finger flexor pulley system, flexor tendon tenosynovitis, and capsulitis/synovitis (1). Several studies have investigated the prevalence and nature of finger injuries in climbers with incidence rates ranging from 30% to 40% (2–4). A study by Schweizer et al. found that finger injuries accounted for over 38% of all acute and overuse climbing injuries (2). Another study by Schöffl et al. found that over 30% of climbing injuries reported occurred in the fingers (3). Similarly, a study by Grønhaug found that over 40% of climbers self-reported experiencing finger injuries (4). A critical review of the incidence and risk factors for finger injuries in rock climbing additionally identified that the fingers are the most common site of injury (5).

Capsulitis/synovitis accounts for approximately 6%–10% of all climbing injuries and is the second most common injury in the finger (3). Despite a few studies that have examined the epidemiology of hand and finger injuries in rock climbers, there is a lack of research on the management of these injuries, particularly capsulitis and synovitis. With capsulitis and synovitis being such common injuries among climbers, it is important to understand what the condition entails to develop effective management strategies.

**Figure 1:**
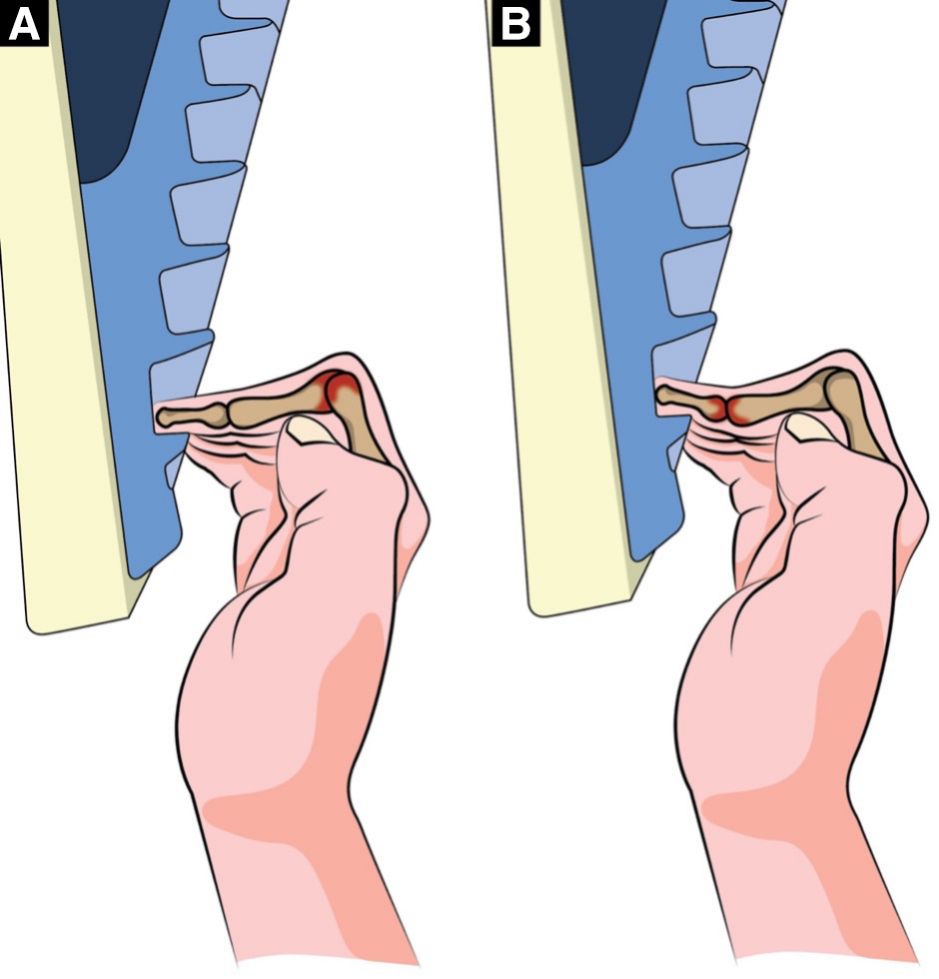
(**A,B**) Left to right. Half and full crimp with the red indicating the major stress regions of each type of crimp.

Capsulitis is described as an inflammatory condition in a joint capsule. Histologic studies have shown chronic fibrosis of the capsule, with the predominant cells involved being fibroblasts and myofibroblasts (6). Synovitis describes the inflammatory histological changes that occur within an affected joint. This includes synovial lining hyperplasia, infiltration of macrophages and lymphocytes, neoangiogenesis, and fibrosis (7). In climbers, capsulitis/synovitis mostly affects the proximal (PIP) and distal interphalangeal joints (DIP) of the fingers. Capsulitis/synovitis in the PIP joint most often occurs from the high peak pressure within the finger PIP joints during the half or full crimp position (Figures 1A,B) (8). The crimp grip in climbing is used when contacting small holds. A half crimp involves flexion of the metacarpophalangeal joint (MCP) and the PIP joint and no hyperextension of the DIP joint (9). A full crimp involves flexion of the MCP and the PIP joint and hyperextension of the DIP joint (9). In addition to compression at the PIP joint during a half crimp, capsulitis/synovitis can occur as well at the DIP joints during full crimping secondary to high forces during DIP hyperextension (Figure 1B). In both cases, the stress on the finger joint is localized to one location rather than being spread across the entire joint surface. The onset of finger capsulitis/synovitis in climbers can be chronic and develop over time from repetitive microtraumas, such as climbing with increased volume or intensity or the increased use of the crimp grip. It can also occur secondary to acute trauma, such as twisting the fingers into a crack, losing footing, or hitting the knuckle against the rock wall. With either mechanism, the climber often presents with edema, stiffness, and a dull ache in the dorsal and/or lateral DIP or PIP joint. Symptoms typically decrease with warming-up and mid-range activity (such as ball squeezes or rice bucket finger curls) similar to clinical reports of osteoarthritis (10). However, although capsulitis/synovitis is commonly present during osteoarthritis, it can also occur in isolation and is typically a precursor to chronic osteoarthritis in climbers (8). The article aims to present a case study of a recreational rock climber with finger joint capsulitis/synovitis and provide a comprehensive rehabilitation program based on a progressive framework that resulted in a full recovery and could serve as a basis for future research and management of similar injuries in rock climbers.

## Methods

A 23-year-old male rock climber, who climbed an average of 3–4 times per week with a combination of indoor climbing during the week and outdoor climbing on the weekend, was evaluated for left fourth digit finger pain in the PIP region. He had 6.5 years of bouldering experience with a pre-injury grade of V8. He reported that, for the first 4–5 years of climbing, he would climb 3 days per week at a moderate intensity and supplement his climbing with core training, weighted pull-ups, shoulder strength exercises, and light training on a fingerboard (hanging bodyweight from the fingers on various depths of edges). The climber reported a history of pain in his finger on and off during that time. He then gradually began increasing the intensity of his climbing from moderate to high. He stopped performing supplemental training exercises but continued fingerboarding with a combination of moderate to maximal hangs (7 s hang followed by 1–3 min rest) and repeater hangs (7–10 s hang followed by 3–5 s rest). In the 6 months, his fingerboard sessions were becoming less structured, and he reported an increased intensity of climbing, which led up to his injury. Although he was feeling gradual discomfort in his finger, he did not fully notice it until during a fingerboard session where he was hanging his body weight on a small edge for 7 s. He discontinued the fingerboard session and attempted to climb the next day, but he still felt finger discomfort. He attempted self-care for several weeks that consisted of decreasing his climbing intensity and stretching his finger into flexion to relieve the pain, but it made the symptoms worse, so he scheduled a physical therapy appointment for an evaluation of the injury. During the evaluation, 2 months after the onset of the injury, he reported moderate finger pain with a severity of 5.5 out of 10 on the visual analog scale (VAS) scale 24 h after climbing with 0 being “no pain” and 10 being “pain as bad as it could possibly be.” He also reported a chronic history of right-sided low back pain with increased intensity 8 weeks prior to the evaluation. Secondary to pain, he was limited to climbing to the grade of V5. He denied any radiating pain or numbness in the hand or fingers.

**Figure 2:**
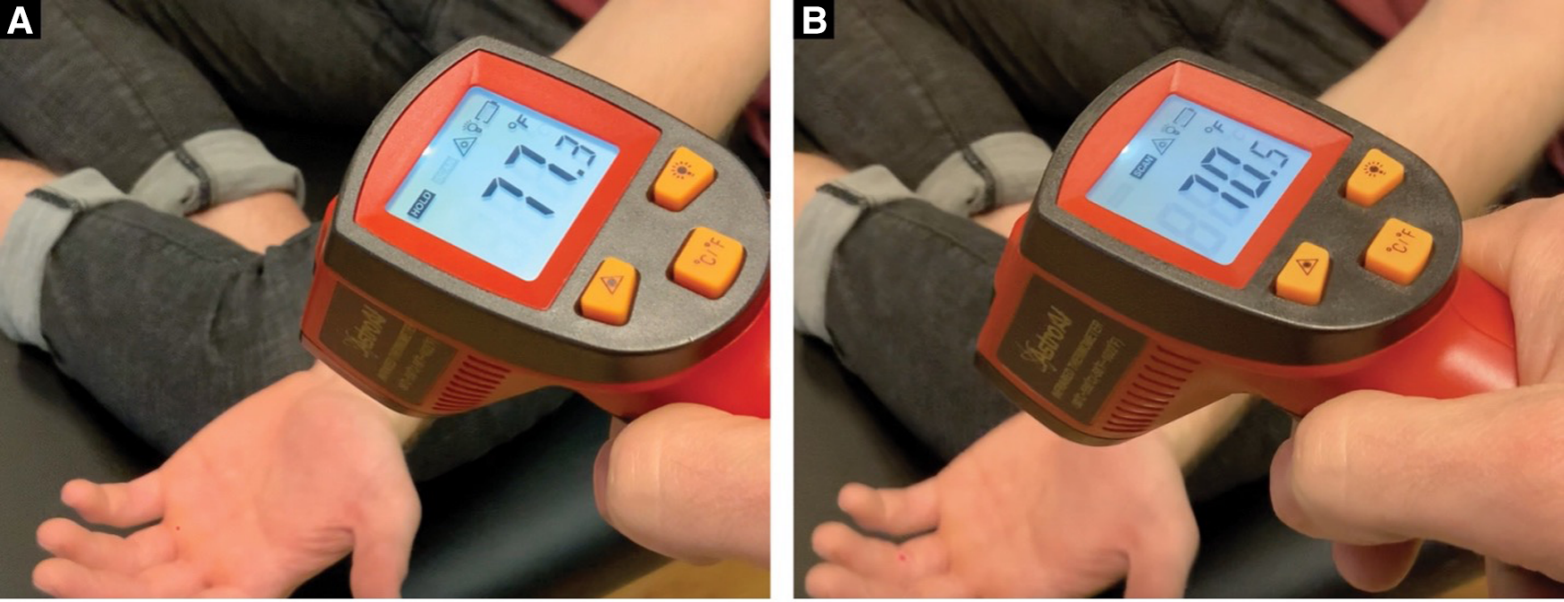
(**A**) Tissue temperature taken at the MCP joint. (**B**) Tissue temperature taken distal to the PIP joint.

**Figure 3:**
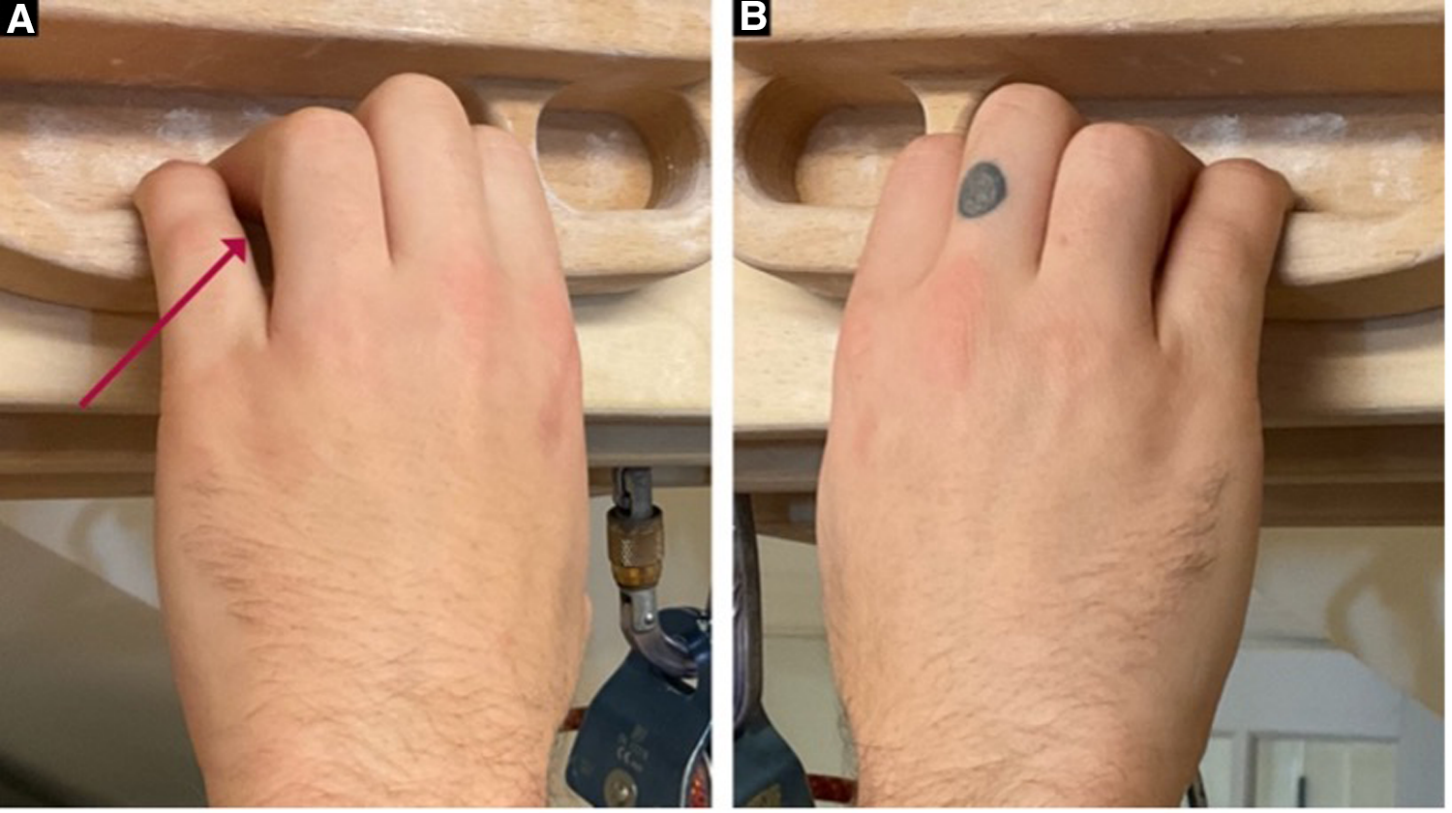
(**A**) Left hand hanging on a fingerboard with fifth digit abduction, MCP flexion, and PIP extension. (**B**) Right hand hanging on a fingerboard for comparison.

**Figure 4:**
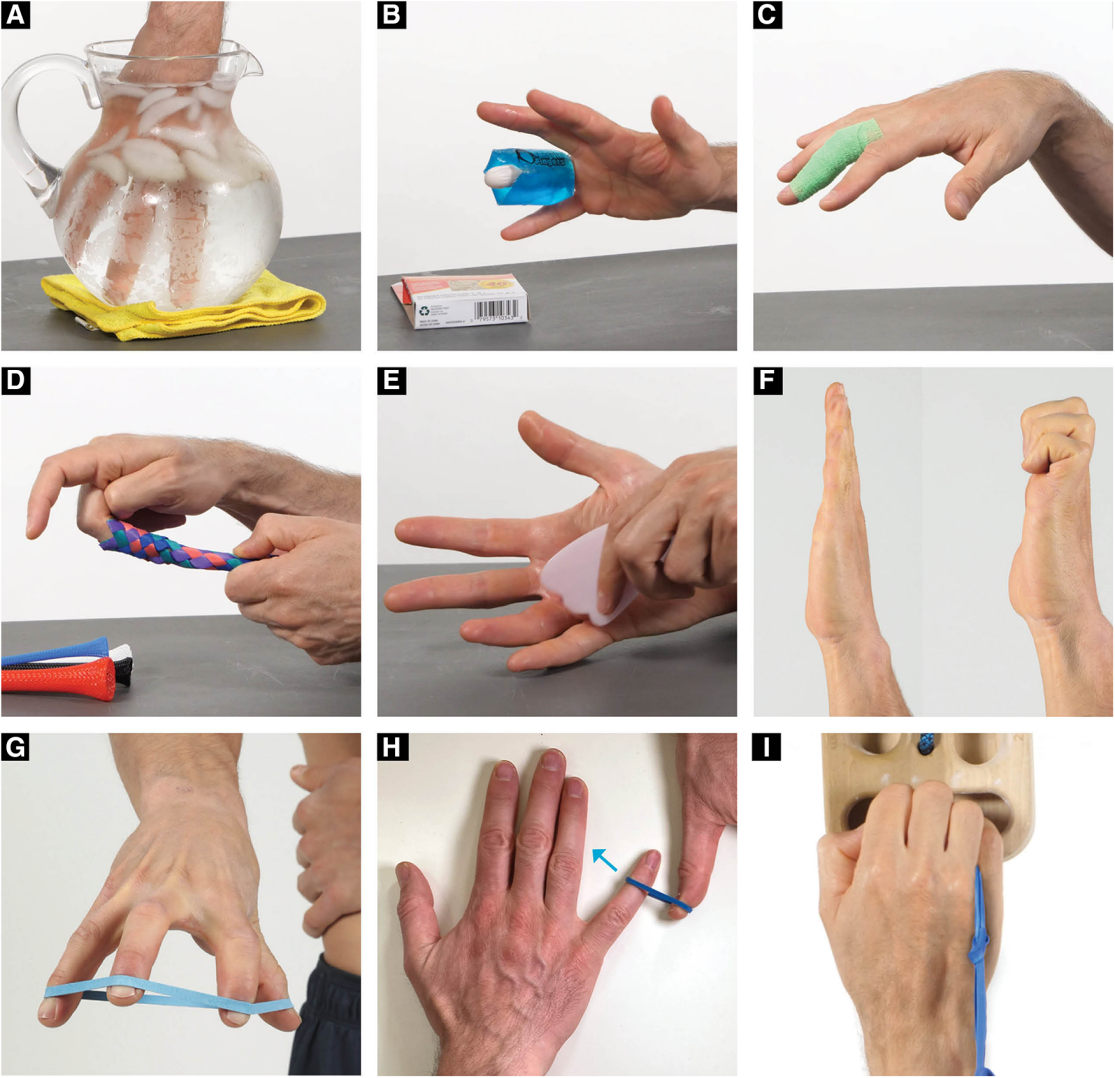
(**A**) Finger icing in water, (**B**) Finger icing with wrap, (**C**) Self-adherent compression bandage wrap range of motion, (**D**) Finger trap oscillations, (**E**) Instrument assisted soft tissue mobilization, (**F**) Hook fist active range of motion, (**G**) Resistance band flicks, (**H**) Open chain palmer interosseous resistance exercises, (**I**) closed chain gripping palmer interosseous resistance exercises.

The clinical examination included finger joint range of motion with overpressure, joint compression/distraction, tissue temperature comparisons (AstroAI Infrared Thermometer) above and below the PIP joint (Figure 2), and movement observation of the climber hanging from their fingertips from a fingerboard (Figure 3). Imaging was not available to use for assessment, so the objective tests and measures were used to make a clinical diagnosis. Based on the subjective reports and objective data gathered, the climber was given a home exercise program based on a rehabilitation framework to unload the affected tissues, improve mobility, increase muscle performance, and retrain climbing movement (11). Interventions consisted of unloading techniques for the first 2 weeks, icing the finger for 5 min (either in an ice bucket or with a cold compressive gel pack) once per day, wrapping the finger in a self-adherent compression bandage wrap or floss band, and performing active range of motion for three sets of 45 s daily (Figures 4A–C). Additionally, for the duration of 6 weeks, the climber was prescribed daily mobility exercises for three sets of 45 s each including oscillatory PIP joint mobilizations (using a finger trap to separate the joint surfaces and blocking the middle phalanx with the thumb), instrument-assisted soft tissue mobilization of the fingers with moderate pressure, and active straight fingers to hook fist range of motion (Figures 4D–F). The climber was also given strength exercises to be performed three times per week including rubber band flicks and palmer interosseous gripping exercises (Figures 4G–I). The climber was told after 6 weeks that he could reduce the frequency of the mobility and strength exercises from once per day to three times per week. The climber was instructed to refrain from climbing and training for a period of 2 weeks. After this time, he was advised to gradually resume their regular climbing and training schedule of 3–4 sessions per week by adjusting the intensity and volume of their sessions. Six weeks following the start of the program, the climber was given permission to climb without restrictions but advised to self-regulate the volume and intensity of their climbing based on their level of comfort. The climber preferred a treatment structure that consisted of an initial session followed by a home exercise program to allow for self-management, rather than repeated sessions.

Exercises were reviewed during the initial evaluation with manual and verbal feedback, and correct exercise performance was confirmed. Detailed videos and written descriptions of the exercises were provided to the climber. During the first 6 weeks of the program, the climber demonstrated high adherence with almost full compliance, as assessed through a subjective report questionnaire that tracked the number of self-therapy sessions completed. However, as symptoms began to improve, the climber self-reported moderate adherence from week 6 to month 12, with no formal monitoring conducted during this period.

The VAS; sports-specific disabilities of the arm, shoulder, and hand (DASH); and patient-specific functional scale (PSFS) were used to monitor patient progress during the initial evaluation, 6-week follow-up, and 12-month discharge. The VAS was given prospectively, and the sports-specific DASH and PSFS were given retrospectively. The VAS scale, ranging from 0 to 10, was used to measure the patient's pain levels 24 h after climbing, providing a validated subjective measure for pain. The sports-specific DASH is a module within the DASH that was utilized to determine the impact of the injury on sports participation, consisting of four questions related to the injury's effects. The scores of each question were averaged to calculate the percentage of disability. The PSFS was used to identify three self-selected activity limitations, including the climber's ability to crimp on boulder problems, project boulder problems at 80% of maximum intensity, and complete a full training session without modification. Each limitation was scored from 1 to 5 based on the patient's ability to perform, and the scores were averaged to calculate a percentage of ability.

## Results

The climber presented with reports of left ring finger pain (dorsal greater than volar) in the region of the PIP joint. He presented with a VAS of 5.5/10, a sports-specific DASH of 69%, and a PSFS score of 0%, 24 h after climbing. During clinical testing, the climber presented with mild swelling of the left fourth PIP joint. Joint overpressure into flexion and extension reproduced 4/10 symptoms and joint distraction decreased symptoms to 0/10 in end-range positions. The climber presented with a 6.8°F decrease in temperature distal to the affected PIP joint (Figure 2) on the left fourth digit and only a 3° change on the right hand. A negative Bunnell–Littler test was used to rule out the involvement of intrinsic muscle stiffness limiting the joint range of motion. Additionally, during movement analysis, it was discovered that when the climber hung from a fingerboard, the climber demonstrated an asymmetric position of the fingers on their left hand. The primary fault was excessive left fifth digit metacarpal phalangeal (MCP) flexion and PIP extension (Figure 3). The climber performed his home exercise program independently as prescribed for 6 weeks. At the 6-week follow-up, the climber presented with a VAS of 1.5/10, sports-specific DASH of 34%, and PSFS score of 43%, 24 h after climbing. He had returned to training and climbing pain-free at his previous grade of V8 cautiously but without restriction. At the 12-month discharge, his symptoms had decreased to 0/10 24 h after climbing, his sports-specific DASH was reduced to 6%, his PSFS improved to 98%, and his climbing ability improved from V5 with pain to V8 pain-free (Table 1).

**Table 1:** Subjective report questionnaire.

Questionnaire	Injury onset	6 weeks	1 year
VAS	5.5/10	1.5/10	0/10
Sports-specific DASH	69%	34%	6%
PSFS	0%	43%	98%
Climbing grade	V5	V8	V8

VAS, visual analog scale (from 0 to 10 reported 24 h after climbing); DASH, disabilities of the arm, shoulder, and hand; PSFS, patient-specific functional scale.

Sports-specific DASH: The DASH module for assessing the impact of an injury on sports participation comprises four questions pertaining to the injury's effects, and the scores from each question are averaged to determine the percentage disability. PSFS: Three self-selected activity limitations were identified for the climber, which included crimping on boulder problems, projecting boulder problems at 80% of maximum intensity, and completing a full training session without modification. These limitations were scored on a scale of 1–5, based on the patient's ability to perform, and the scores were averaged to calculate a percentage of ability.

## Discussion

**Figure 5:**
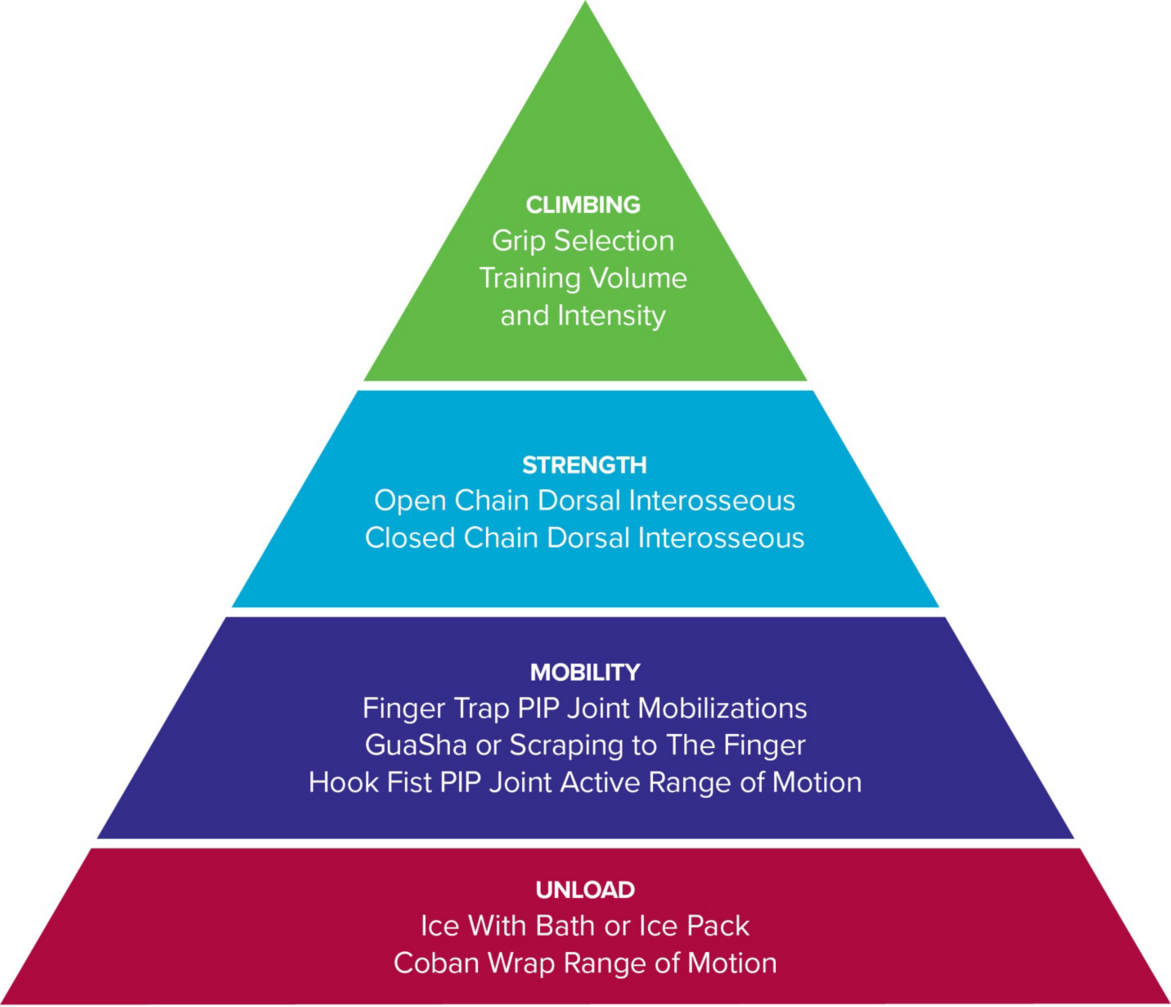
Organization of rehabilitation into a framework.

A comprehensive rehabilitation program was developed based on the concepts of a progressive framework that included unloading the affected tissues, increasing mobility, improving muscle performance, and addressing climbing movement and training (Figure 5) (9, 11).

The unloading techniques included two methods (ice and active compression) to reduce inflammation and improve mobility in the affected finger. Cold compression has been shown to be effective in decreasing pain and improving mobility in patients with inflammatory conditions such as arthritis (12). Additionally, the climber was told to wrap the digit with a self-adherent compression bandage wrap (less aggressive) or a floss band (more aggressive) and to perform finger flicks. It has been shown that floss band wrapping of peripheral joints can increase joint range of motion, manage pain, and reduce muscle tightness (13). This active compression method was chosen over the use of compression gloves since the research on the effectiveness of passive prolonged compression with gloves in cases of rheumatoid arthritis and hand osteoarthritis remains inconclusive (14).

The rationale behind the mobility techniques was to restore pain-free mobility and reduce joint capsule tissue tension in end-range positions. Finger traps were used during oscillations to allow the climber to obtain a better grip to mobilize the affected finger. The climber was instructed to place the finger in the resting position and to perform three sets of 45 s (15). Additionally, the climber was instructed to perform gentle instrument-assisted soft tissue mobilization to the fingers as this technique has been shown to be effective in improving pain and patient-reported function (16). After the climber improved their joint mobility with the finger trap oscillations and improved adjacent tissue mobility with instrument-assisted soft tissue mobilization, the climber was told to perform an active range of motion of the PIP joint from a straight hand to a hook fist to use the range of motion that they had just gained.

It has been shown in research that there is a deficit in finger extensor strength ratios to finger flexors in rock climbers when compared to non-climbers (17). It is proposed that climbers may benefit from finger extensor training to balance the strength of the muscles that move the fingers. Additionally, the finger extensor tendons (extensor digitorum communis extensor indices and extensor digiti minimi) play a role in micro-adjusting finger position while gripping. In particular, extensor digiti minimi can extend the fifth digit at the MCP joint while gripping to help the climber reduce the excessive MCP flexion of his fifth digit that was observed during fingerboarding. The climber also presented with decreased temperature in the affected finger distal to the PIP joint when compared to the other side which likely was a result of the decreased circulation distal to the PIP injury. While there are no standardized normative values for an acceptable tissue temperature difference to reflect decreased circulation, the fact that commercially available infrared thermometers have been validated for measuring skin surface temperature associated with deep and surrounding wound infections still makes the information useful in the context of the climber's presentation (18). For these reasons, rapid active finger movements into a rubber band (rather than isometric) were prescribed to strengthen the finger extensor tendons and improve finger circulation prior to climbing with the proposed mechanism of creating a muscle/tendon pump to promote increased tissue temperate through the arterial system and recirculation of the edema through the venous system.

Based on the climber's gripping on the fingerboard, additional exercises were added to improve open and closed kinetic chain finger positions with an emphasis in the fifth digit positioning. Since the climber's fifth digit was abducted, flexed at the MCP joint, and extended at the PIP joint, this can place greater amount of stress on the fourth digit secondary to the loss of lateral support from the pinky. Greater loads while crimping have been shown to increase joint forces and this can potentially lead to joint capsulitis/synovitis (8, 19). Based on this hypothesis, both open chain exercises for the palmar interosseous muscles (in particular the 3rd palmer interosseous) and closed kinetic chain exercises while gripping were given. Both open and closed kinetic chain finger exercises were hypothesized to improve muscle performance (open kinetic chain) and movement coordination (closed kinetic chain) during gripping to reduce joint torsion and PIP loading.

**Figure 6:**
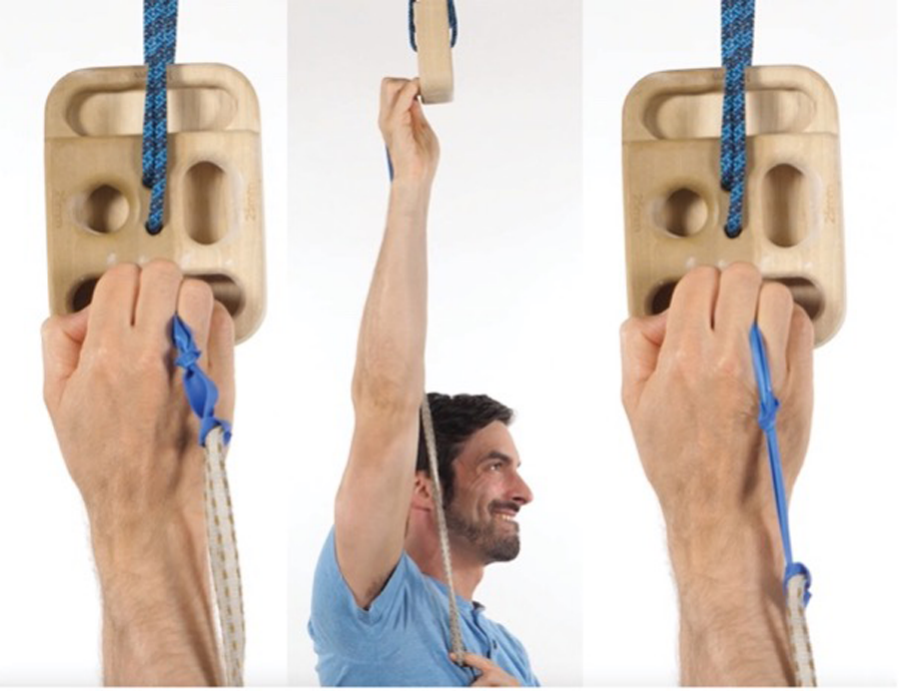
Closed kinetic chain interossei exercise demonstrated between the third and fourth digit. The climber performed this same exercise but with the band between the fourth and fifth digit.

The open kinetic chain exercise was performed by having the climber press their pinky and/or ring finger into a resistance band. The closed kinetic chain exercise was a novel exercise that involved the climber placing a rubber band (connected to a 2 ft string or piece of climbing webbing) between the fourth and fifth digit and squeezing the band tightly as it is pulled downward while trying not to allow the band to slip (Figure 6).

Since the climber presented only with mild symptoms, they were recommended to discontinue climbing and training for 2 weeks, followed by a gradual return to full climbing and training intensity while using the crimp grip sparingly. He was encouraged during his return to full climbing and training to focus the movement modification of engaging the fifth digit while crimping. Additionally, since the climber showed signs of decreased temperature to the affected finger, he was given a comprehensive warm-up to perform prior to climbing to improve tissue temperature and prepare the fingers for loading.

Limitations of this case include a sample size of one and lack of imaging to confirm the diagnosis of synovitis/capsulitis. Moreover, there is a paucity of research on the average recovery time and timeline for return to sports following joint synovitis and capsulitis in rock climbers. Therefore, while the climber in this study made steady progress and was able to return to his pre-injury grade of climbing, the effectiveness of the described rehabilitation framework cannot be accurately evaluated or measured in this single study due to the absence of comparable data in the existing literature. The retrospective administration the sports-specific DASH and PSFS at the 12-month follow-up may have introduced recall bias in this study. However, the climber maintained meticulous weekly training logs and notes that were utilized during the evaluation process to minimize the impact of potential bias.

## Conclusion

The results of this study suggest that the use of a progressive framework, which includes unloading, mobility, strength, and movement training, holds promise for rehabilitating early-stage finger joint capsulitis/synovitis in rock climbers over short-term (6 weeks) and long-term (1 year) periods. While this study cannot definitively conclude the effectiveness of the progressive framework without comparisons to other treatment approaches, the findings are encouraging and warrant further investigation in future research.

## Data Availability

The original contributions presented in the study are included in the article/Supplementary Material, further inquiries can be directed to the corresponding author.
