# Supplementary figures and images for: Clinical management of finger joint capsulitis/synovitis in a rock climber
Source: Front Sports Act Living. 2023 May 25;5:1185653. doi: 10.3389/fspor.2023.1185653 (PMC10266264; doi:10.3389/fspor.2023.1185653)

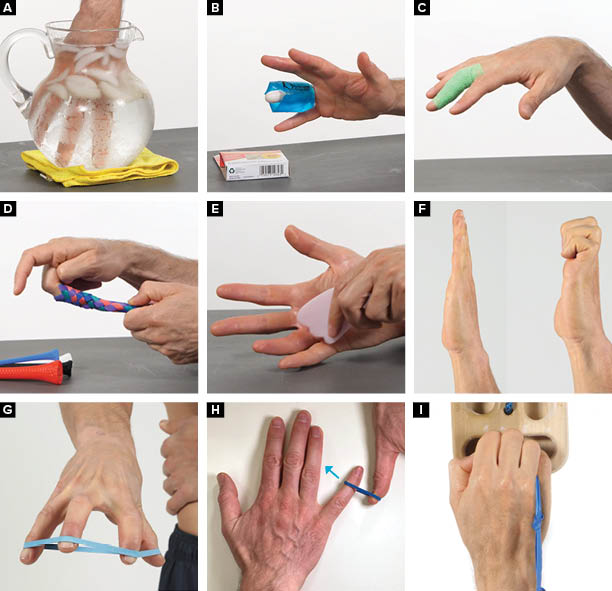

Supplement: Supplementary file 1 [file Image1.jpg]

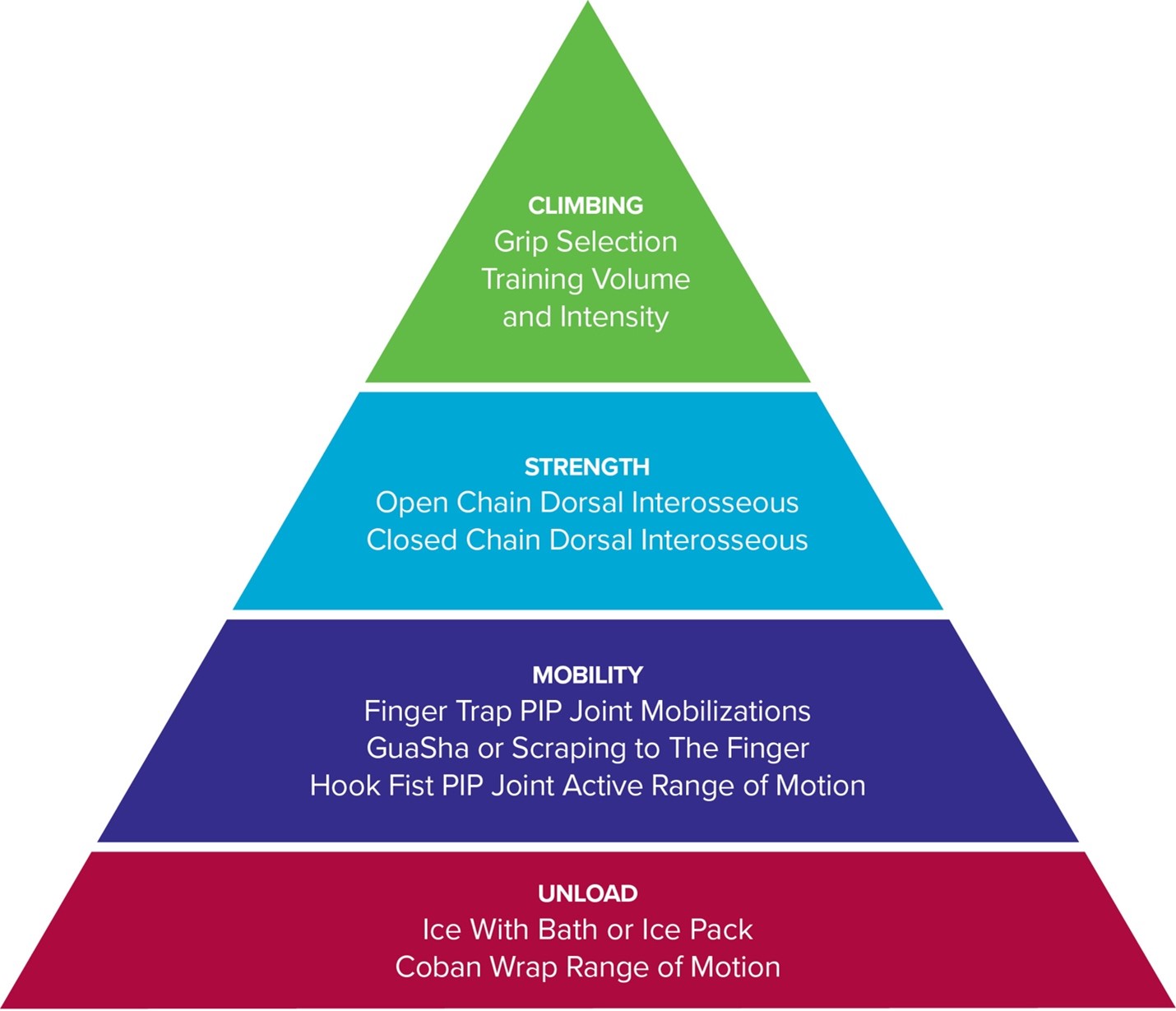

Supplement: Supplementary file 2 [file Image2.jpg]
